# Validation of an integrated pedal desk and electronic behavior tracking platform

**DOI:** 10.1186/s13104-016-1882-0

**Published:** 2016-02-09

**Authors:** John M. Schuna, Catrine Tudor-Locke, Mahara Proença, Tiago V. Barreira, Daniel S. Hsia, Fabio Pitta, Padma Vatsavai, Richard D. Guidry, Matthew R. Magnusen, Amanda D. Cowley, Corby K. Martin

**Affiliations:** Pennington Biomedical Research Center, 6400 Perkins Road, Baton Rouge, LA 70808 USA; School of Biological and Population Health Sciences, Oregon State University, Corvallis, OR USA; Department of Kinesiology, University of Massachusetts Amherst, Amherst, MA 01003 USA; Laboratório de Pesquisa em Fisioterapia Pulmonar (LFIP), Departamento de Fisioterapia, Universidade Estadual de Londrina (UEL), Londrina, PR Brazil; CAPES Foundation, Ministry of Education of Brazil, Brasília, DF 70040-020 Brazil; Department of Exercise Science, Syracuse University, 900 S. Crouse Ave., Syracuse, NY 13210 USA; Vinformatrix, Baton Rouge, LA USA; St. James Technologies, Baton Rouge, LA USA

**Keywords:** Physical activity, Sedentary behavior, Workplace, Exercise, Pedal desk

## Abstract

**Background:**

This study tested the validity of revolutions per minute (RPM) measurements from the Pennington Pedal Desk™. Forty-four participants (73 % female; 39 ± 11.4 years-old; BMI 25.8 ± 5.5 kg/m^2^ [mean ± SD]) completed a standardized trial consisting of guided computer tasks while using a pedal desk for approximately 20 min. Measures of RPM were concurrently collected by the pedal desk and the Garmin Vector power meter. After establishing the validity of RPM measurements with the Garmin Vector, we performed equivalence tests, quantified mean absolute percent error (MAPE), and constructed Bland–Altman plots to assess agreement between RPM measures from the pedal desk and the Garmin Vector (criterion) at the minute-by-minute and trial level (i.e., over the approximate 20 min trial period).

**Results:**

The average (mean ± SD) duration of the pedal desk trial was 20.5 ± 2.5 min. Measures of RPM (mean ± SE) at the minute-by-minute (Garmin Vector: 54.8 ± 0.4 RPM; pedal desk: 55.8 ± 0.4 RPM) and trial level (Garmin Vector: 55.0 ± 1.7 RPM; pedal desk: 56.0 ± 1.7 RPM) were deemed equivalent. MAPE values for RPM measured by the pedal desk were small (minute-by-minute: 2.1 ± 0.1 %; trial: 1.8 ± 0.1 %) and no systematic relationships in error variance were evident by Bland–Altman plots.

**Conclusion:**

The Pennington Pedal Desk™ provides a valid count of RPM, providing an accurate metric to promote usage.

## Background

Evidence suggests that protracted periods of sedentary behavior, for example as a result of occupational demands for seated computer-based work, are associated with reduced total energy expenditure [1], increased abdominal obesity [2], weight gain [3], and increased cardiometabolic risk [4]. Traditional approaches to workplace wellness interventions intended to counteract these effects typically provide access to fitness facilities or exercise sessions (group or individual) during lunch and other work breaks [5]. These approaches have been nominally effective [5] in part because they necessarily shift the requirement of compensating for long periods of low occupational energy expenditure to a diminishing amount of personal time eroded by competing obligations and priorities [6]. Further attempts to engage the office worker in additional workplace physical activity include prompts to increase stair use [7]. Unfortunately, the effectiveness of workplace stair-climbing interventions appears to be limited and short-lived [8].

Innovatively, active workstation alternatives to conventional seated-desk and computer configurations have emerged as potentially effective strategies for replacing workplace sedentary behaviors with light intensity (e.g., 1.6–2.9 METs) and tolerable non-exercise physical activity, thus elevating daily energy expenditure meaningfully if used frequently and for a sufficient duration [9]. Active workstations proposed include treadmill desks [10, 11] and seated pedal/cycle/elliptical desks [12–14]. Reports of typical workplace treadmill desk usage (on days that they are used) range from 30 to 45 min/day [15–17] to as much as 90–100 min/day [18, 19] in select user groups. In two separate intervention studies, Carr et al. [21] reported that workers used under-desk pedal devices for 23 min/day [20] (on days that they were used) to 31 min/day. It is important to emphasize that use of these active workstations represents light intensity physical activity, which is below what is typically recommended in federal physical activity guidelines [22]. Active workstations are intended to replace sedentary behavior, not exercise [9].

As part of the continued development of our own active workstation alternative, the Pennington Pedal Desk™, and in preparation for deploying a workplace intervention centered around it, we developed sensing hardware and accompanying software to facilitate users’ monitoring of their pedal desk usage characteristics. Data generated through usage of the pedal desk is received and initially processed by a local software agent that we have named the Pedal Desk tracker. Once a user has been authenticated by providing a valid username and password, the tracker will keep a local database of data received in addition to transmitting data to the network server for cloud storage (when a network connection is available). These data are available to the end-user in real time and in aggregate (i.e., daily, weekly, monthly) via a custom-designed graphical user interface (GUI) which is a part of the Pedal Desk tracker software package. In addition to end-user access, administrators and interventionists are able to view all participants’ data via a secure login, which facilitates extrinsic support for behavior change. Pedal Desk tracker software was designed for sole use with the Pennington Pedal Desk™ and is not currently compatible with other pedal desks or exercise bikes. However, Pedal Desk tracker software can be installed on multiple computer platforms including Windows, Mac OS X, and Linux.

Tracking of use is necessary to monitor and support behavior change in the workplace. Although some active workstation alternatives provide a real-time digital display of time accumulated [12, 16] and/or distance accrued [12], a method of recording and summarizing a history of use is an important biofeedback feature. Usage data can be tracked as time (e.g., min/day) via a built in sensor to detect motion and start a time clock. Further, accumulated time use over the course of a day could be easily tallied. In the same way, pedal revolutions can be tracked over set time periods (revolutions per minute, or RPM) or even tallied and presented as a cumulative total, for example revolutions per day (revs/day). Presented in this unique format, revs/day is similar to established approaches to tracking and motivating steps/day [23, 24].

Previous work by Elmer and Martin [14] evaluated power estimates provided by a pedal desk; although the accuracy of the pedal desk’s pedal rate measurement system was not reported. Another investigation by Rovniak et al. [25] assessed the accuracy of revolution measurements from a compact elliptical device intended for under-desk deployments. Measured revolutions from the compact elliptical device were reported to perfectly agree (100 % agreement) with those counted manually via direct observation during three consecutive 15 revolution trials. However, the validity of pedal rate measurement technologies used in active workstation tracking systems has not been evaluated over longer durations (i.e., ≥20 min) likely to be encountered in actual workplace settings.

The purpose of this paper is to describe our preliminary work developing and validating the two-tier tracking system for monitoring usage of the pedal desk. As noted earlier, usage is quantified and monitored by tracking RPM, and the validity of RPM measurements from the pedal desk were tested during this research project. We hypothesized that RPM measurements from the pedal desk during a simulated working experience would be equivalent to those provided by an accelerometer-based cadence sensor (i.e., Garmin Vector, Garmin^®^, USA).

## Methods

### Regulatory

The study was approved by the Pennington Biomedical Research Center’s Institutional Review Board. All participants provided written informed consent prior to commencement of any assessment procedures.

### Development of the pedal desk and tracking technology

**Fig. 1:**
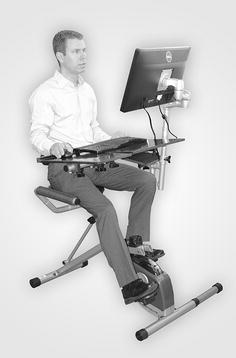
Pedal Desk

The Pennington Pedal Desk™ (Fig. 1) is a single and integrated piece of office-ready furniture that includes a height-adjustable desktop and opportunities to accommodate keyboard and monitor positioning preferences. The desktop is fully maneuverable; it swings, tilts, and shifts forwards and backwards. The pedaling mechanism is belt driven and therefore quiet. Revolutions are counted when a sensor located in the pedaling mechanism is activated by a magnet secured to the flywheel. Data corresponding to each pedal revolution (timestamp) are transmitted to a computer via USB cable. Resistance to the pedal desk’s flywheel is provided by a magnetic braking mechanism and is not adjustable. The level of flywheel resistance (≈0.30 kiloponds) was chosen to facilitate long-term pedal desk usage, without undue fatigue, as pedal rates of 30–90 RPM would result in respective power outputs of approximately 12–36 W. The aforementioned range of power outputs falls at or below the 30–50 W range consistent with stationary cycling of “very light to light effort” [26].

**Fig. 2:**
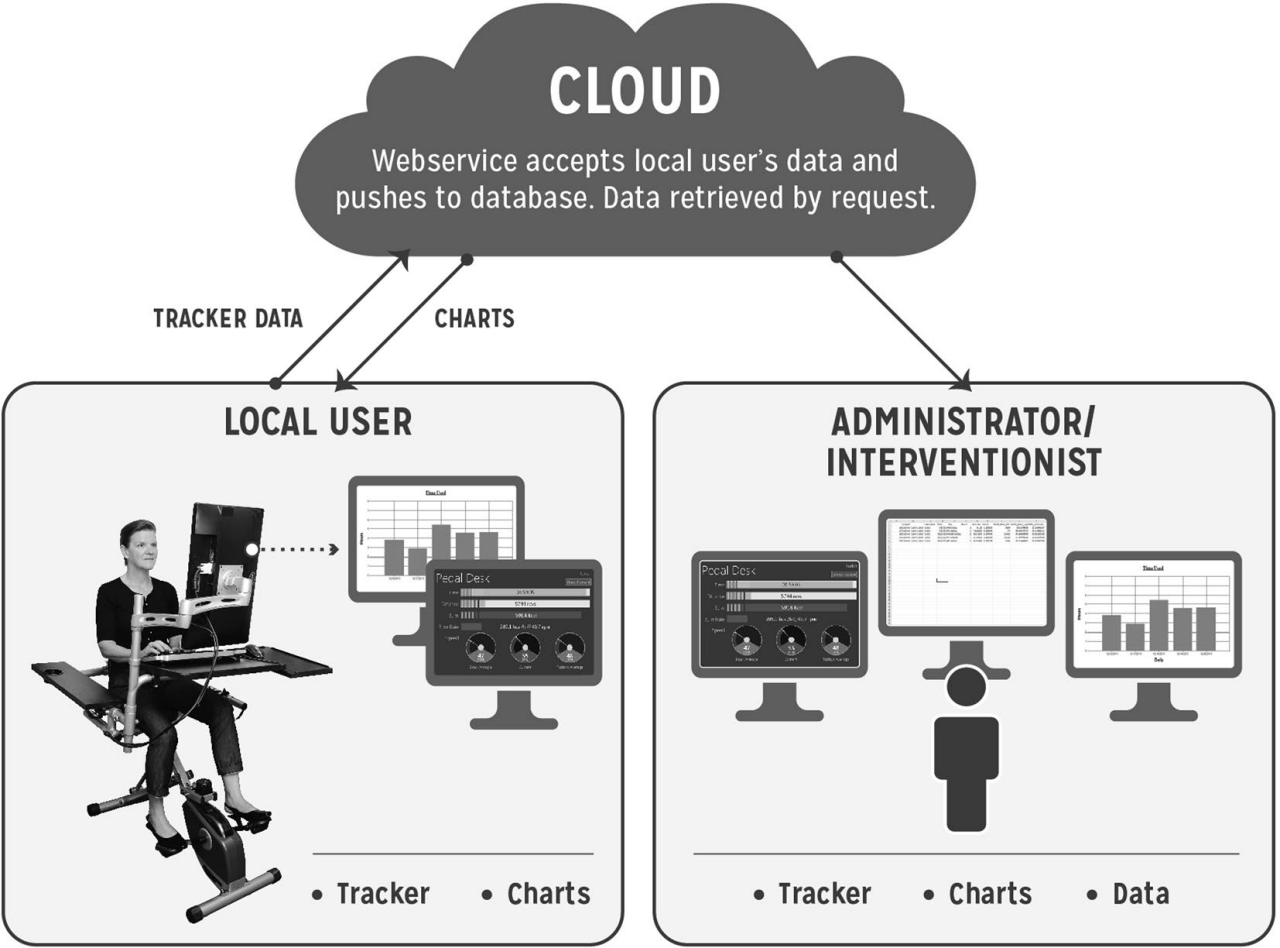
Pedal Desk and Pedal Desk tracker architecture

The pedal desk is part of a comprehensive behavior monitoring and tracking system (see Fig. 2 for the overall system architecture). As previously mentioned, the direct outputs available from the tracking technology (Java-based Pedal Desk tracker GUI) include duration of use and RPM, which are extracted from a SQL database containing one timestamped observation for every recorded pedal revolution. Real time duration of use is displayed as a daily running total and is continually updated by adding the lapsed time between the current and preceding pedal revolutions as long as this lapsed time is <15 s. Similarly, real time RPM values are updated on a pedal-by-pedal basis and also calculated using the lapsed time (s) between the current and preceding pedal revolutions (RPM = [1/lapsed time] × 60). Users may also extract summary totals (duration of use) and averages (RPM) over daily, weekly, and monthly time spans. Duration of use summary totals are quantified in minutes by (1) classifying collected pedal-by-pedal data into distinct bouts (i.e., time sequences of pedaling separated by ≥15 s), (2) calculating the duration of each bout (ending timestamp − starting timestamp), and (3) summing the duration of all bouts over the specified interval (e.g., daily, weekly, monthly). RPM summaries are quantified by (1) counting the number of observations within each bout, (2) summing the number of observations over the specified time interval (e.g., daily, weekly, monthly), and (3) dividing the total number of observations by the associated total duration of bouts in minutes. Further summarization and analysis of captured data can be conducted by querying the SQL database which stores all collected data from the pedal desk.

### Procedures

Participants consisted of 44 full-time Pennington Biomedical Research Center employees (73 % female; 39 ± 11.4 years-old; BMI 25.8 ± 5.5 kg/m^2^, 28.8 ± 9.6 % body fat [mean ± SD]) who responded to an internal email advertising the study and described their typical working day as primarily sitting “most of the time.” Additional inclusion criteria were being 21–65 years of age and familiarity with using a computer to compose emails and search the internet. Exclusion criteria were being: (1) >250 pounds (a limitation of the prototypical pedal desk design), (2) pregnant or having a pacemaker or metal joint replacement (a restriction related to body composition measurement using bioelectrical impedance), and (3) unable to perform a pedaling-based movement. Participants were shoeless for all anthropometric measurements. Height was measured with a wall mounted stadiometer and weight and body fat percentage were measured using a Tanita Body Composition Analyzer (SC-240).

Following familiarization with the Pedal Desk, participants pedaled at a self-selected pace for an approximate 20 min trial that included guided computer-based tasks (internet search of a randomly generated topic, composing and sending an email about the topic, and completing an on-line questionnaire). In addition to RPM measurements obtained via the pedal desk, Garmin Vector power meter pedals (accelerometer-based cadence sensor) linked with a Garmin EDGE 510 GPS bicycle computer (Garmin^®^, USA) were used to continuously quantify RPM during all testing. Participants were not presented with any digital feedback at any time from either the Pedal Desk tracker software or the Garmin EDGE bicycle computer. A video camera recorded a subsample (n = 9) of participants’ pedaling actions.

### Data processing

Data corresponding to every pedal revolution captured from the pedal desk during each trial were downloaded from a server-based SQL database following protocol completion. Second-by-second data from the Garmin Vector were extracted from the Garmin EDGE 510 GPS using GoldenCheetah, version 3.0, an open-source software program. The accumulated data from both sources were merged on matched timestamps and summarized (averaged) to coarser resolutions at the minute-by-minute and trial level. Video recordings were viewed (direct observation) post-testing and a direct count of RPM was distilled for each minute. Pedal revolutions were counted by identifying the pedal crank’s starting location at the beginning of each minute and subsequently counting the number of times the crank eclipsed this point over the following 60 s.

### Statistical analyses

To validate the accuracy of RPM measurements obtained via the pedal desk, we conducted analyses in a two-step process. First, we established the validity of the commercially available Garmin Vector’s RPM measurements relative to the criterion of directly observed RPM (the gold standard) in a subsample of nine participants, as no validation studies evaluating the Garmin Vector have been published to date. Second, we evaluated the validity of RPM measurements from the pedal desk relative to the Garmin Vector in 41 of the original 44 participants (three participants’ data were lost, one due to Garmin Vector malfunction and two to pedal desk equipment malfunction).

All statistical analyses were performed using SAS 9.4 (SAS Institute LLC, Cary, NC, USA). Summary statistics were computed to describe RPM data collected from all sources (i.e., direct observation, Garmin Vector, pedal desk) during the testing trial. Validity of the Garmin Vector and pedal desk in measuring RPM relative to their respective criterions (direct observation via video and Garmin Vector, respectively) at both the minute-by-minute and trial level was assessed using equivalence testing [27]. Assuming the pedal desk would be used at a mean self-selected pedaling rate of approximately 50 RPM [14], we defined an a priori equivalence margin for the mean difference (criterion method − test method) between −1.5 and 1.5 RPM. As such, the boundaries of this equivalence margin correspond to a maximum allowable mean difference of no >3 %, which was deemed acceptable by the research team at the study’s outset. Confidence intervals (95 %) for the mean difference between methods were then constructed and compared against the boundaries of the defined equivalence margin. If the confidence interval for the mean difference was completely contained between −1.5 and 1.5 RPM (> −1.5 RPM at α = 0.025 and <1.5 RPM at α = 0.025; family-wise α < 0.05 for each pair of comparisons), the mean values for the two methods were deemed equivalent. Statistical comparisons of mean differences against the boundaries of the defined equivalence margin were performed using linear mixed-effects models (PROC MIXED; participant included as a random effect) for minute-by-minute data and *t* tests (PROC TTEST) for trial level data. To provide further context describing the relationships between methods, we quantified Pearson product-moment correlations, mean absolute percent error (MAPE; [absolute error/criterion RPM] × 100), and the percentage of observations with errors of <1, 3, and 5 RPM. Bland–Altman plots were also constructed to assess between method agreement [28] while accounting for the data’s replicate structure (linked-replicates) when defining the limits of agreement for minute-by-minute plots [29].

## Results

**Fig. 3:**
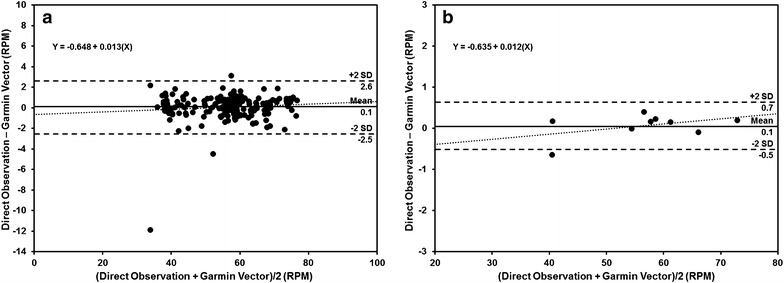
Bland–Altman plots displaying agreement in RPM measurement between direct observation and the Garmin Vector (n = 9). Data are presented in panels: **a** minute-by-minute RPM, **b** mean RPM per trial (≈20 min). *Solid lines* represent the mean bias, *dashed lines* represent the 95 % limits of agreement, and *dotted lines* represent the fitted regression line

Per study design, duration of use was approximately 20 min per trial (20.5 ± 2.5 min) as captured by the pedal desk. Mean values of concurrently measured RPM from both direct observation and the Garmin Vector in the subsample of nine participants are displayed in Table 1. Observed differences in mean RPM between direct observation and the Garmin Vector were negligible (≈0.1 RPM) and the 95 % confidence intervals for mean differences at the minute-by-minute and trial level were completely contained between −1.5 and 1.5 RPM (all *p* < 0.025). The Garmin Vector was highly correlated with direct observation (minute-by-minute: r = 0.99, trial: r = 0.99) and produced mean RPM estimates within ±3 RPM of the criterion more than 98 % of the time. The observed MAPE values for the Garmin Vector relative to direct observation were small (minute-by-minute: 1.5 %, trial: 0.5 %). No substantial biases or discernable relationships were evident in the Bland–Altman plots (Fig. 3) as the slope of the fitted regression line was not significantly different from 0 at the minute-by-minute or trial level (all *p* > 0.05). Additionally, the 95 % limits of agreement were narrow (minute-by-minute: −2.5 to 2.6 RPM; trial: −0.5 to 0.7 RPM).

**Table 1:** Comparison of RPM measurements from direct observation and the Garmin Vector (n = 9)

	Direct observation^a^ (RPM)	Garmin vector (RPM)	Mean difference (RPM)	Mean absolute percent error (%)	<1 RPM error (%)	<3 RPM error (%)	<5 RPM error (%)
Minute-by-minute	56.4 ± 0.8	56.3 ± 0.8	0.1 ± 0.1*	1.5 ± 0.2	78.8	98.4	99.5
95 % CI	(54.8, 58.0)	(54.8, 57.9)	(−0.1, 0.3)	(1.0, 2.0)			
Trial^b^	56.5 ± 3.6	56.5 ± 3.5	0.1 ± 0.1*	0.5 ± 0.2	100.0	100.0	100.0
95 % CI	(48.3, 64.7)	(48.4, 64.6)	(−0.2, 0.3)	(0.1, 0.8)			

Values are presented as mean ± SE unless otherwise noted

*RPM* revolutions per minute

* Significantly > −1.5 and <1.5 at *p* < 0.025

^a^Criterion measure

^b^RPM measurements for trial level data were averaged over an approximate 20 min period

**Fig. 4:**
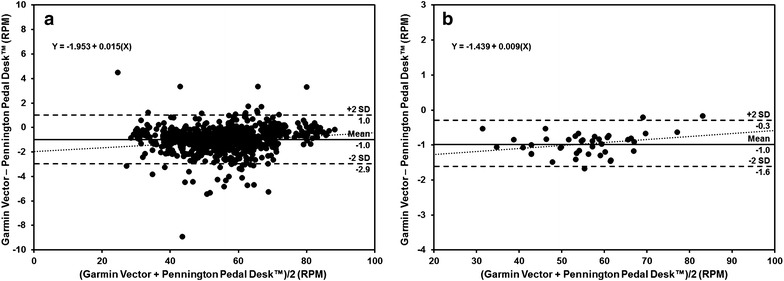
Bland–Altman plots displaying agreement in RPM measurement between the Garmin Vector and the Pennington Pedal Desk™ (n = 41). Data are presented in panels: **a** minute-by-minute RPM, **b** mean RPM per trial (≈20 min). *Solid lines* represent the mean bias, *dashed lines* represent the 95 % limits of agreement, and *dotted lines* represent the fitted regression line

Summary statistics of concurrently detected RPM from both the Garmin Vector and the pedal desk in the sample of 41 participants are presented in Table 2. Observed differences between the Garmin Vector and the pedal desk were small (≈1.0 RPM), yet larger in magnitude than the mean difference between direct observation and the Garmin Vector. However, 95 % confidence intervals for the mean difference at both the minute-by-minute and trial level were contained within the boundaries of the defined equivalence margin (−1.5 to 1.5; all *p* < 0.025). Correlations between the Garmin Vector and the pedal desk were strong (minute-by-minute: r = 0.99; trial: r = 0.99) and the pedal desk produced RPM estimates within ±3 RPM of the Garmin Vector criterion more than 96 % of the time. Observed MAPE values for the pedal desk relative to the Garmin Vector were small (minute-by-minute: 2.1 %, trial: 1.8 %). Bland–Altman plots assessing agreement between the Garmin Vector and pedal desk are depicted in Fig. 4. No identifiable pattern of heteroscedasticity was evident in the plots and the slope of the fitted regression line was not significantly different from 0 at the minute-by-minute or trial level (all *p* > 0.05). In addition, the 95 % limits of agreement for the mean difference were narrow (minute-by-minute: −2.9 to 1.0 RPM; trial: −1.6 to −0.3 RPM).

**Table 2:** Comparison of RPM measurements from the Garmin Vector and the Pennington Pedal Desk™ (n = 41)

	Garmin vector^a^ (RPM)	Pedal desk (RPM)	Mean difference (RPM)	Mean absolute percent error (%)	<1 RPM error (%)	<3 RPM error (%)	<5 RPM error (%)
Minute-by-minute	54.8 ± 0.4	55.8 ± 0.4	−1.0 ± 0.1*	2.1 ± 0.1	55.9	96.7	99.5
95 % CI	(54.1, 55.6)	(55.0, 56.5)	(−1.1, −0.9)	(2.0, 2.2)			
Trial^b^	55.0 ± 1.7	56.0 ± 1.7	−1.0 ± 0.1*	1.8 ± 0.1	53.7	100.0	100.0
95 % CI	(51.6, 58.4)	(52.6, 59.3)	(−1.1, −0.9)	(1.6, 2.1)			

Values are presented as mean ± SE unless otherwise noted

*RPM* revolutions per minute

* Significantly > −1.5 and < 1.5 at *p* < 0.025

^a^Criterion measure

^b^RPM measurements for trial level data were averaged over an approximate 20 min period

## Discussion

The pedal desk is part of a fully automated tracking and intervention system that encompasses the user, the pedal desk, and the Java-based Pedal Desk tracker software. As part of the evaluation of this system, and in agreement with our primary hypothesis, the results presented herein demonstrate that the pedal desk provides an accurate count of pedal revolutions, expressed over time as RPM, compared to the Garmin Vector criterion. Despite a tendency to slightly overestimate RPM values, outputs from the pedal desk were highly correlated with the Garmin Vector and the absolute magnitude of the observed error was small (≈1.0 RPM), well within our predefined acceptable error range (−1.5 to 1.5 RPM). As such, the observed bias in RPM measurement demonstrated by the pedal desk is of little practical significance to real-world applications (e.g., usage >30 min/day) for which it is intended to be used.

Our results are in agreement with a previous report by Rovniak et al. [25] demonstrating that an under-desk elliptical device had excellent revolution counting accuracy. However, and in contrast to our results, the validation described by Rovniak et al. [25] was not conducted with actual participants and only involved three consecutive trials of 15 revolutions. Moreover, no description of the elliptical device’s revolution counting mechanism was provided by the authors. Therefore, the results presented herein detailing the accuracy of the pedal desk’s RPM measurements during a simulated working experience are novel and we know of no other study that has reported similar data. As such, our comparative discourse is necessarily limited. Nonetheless, this initial validation step was necessary to lay the foundation for future research focused on actual pedal desk usage patterns in contemporary workplaces.

Upon inception of this project, we evaluated physical activity behavior tracking strategies to inform development of the Pedal Desk tracker software and to determine which data outputs would be best to track. Pedometers and other motion tracking devices provide direct behavioral feedback to the user as a running daily tally of steps taken [23]. Although manufacturers have applied various algorithms to these simple count data to extrapolate distance traveled and/or energy expenditure, both types of manipulations are known to introduce error related to underlying assumptions [30]. Therefore, tracking the simple raw step count has surfaced as the most common and translatable metric for monitoring ambulatory behavior using both research and commercial grade devices. As evidence of its widespread acceptance, the American College of Sports Medicine included a target number of steps/day in their most recent position paper summarizing physical activity recommendations for cardiorespiratory, musculoskeletal, and neuromotor fitness in apparently healthy adults [31]. We therefore aimed to measure a simple count of pedal revolutions or revs, expressed over time as RPM.

Cyclists typically track their on-bicycle time (from a watch or other time piece) and distance traveled (inferring from a known measured route). They may track their RPM at any specific time point by simply counting pedal revolutions over a set time interval. They may also invest in additional technology (e.g., power and cadence sensors) to more directly measure distance traveled (from a product of counted wheel revolutions and wheel diameter), monitor power, and estimate energy expended. Although use of the pedal desk is obviously reminiscent of riding a bicycle outdoors, distance is not as clear cut an output for a pedal desk user as for a cyclist. The pedal desk is stationary, so linear distance traveled is an imaginary construct. Further, although a cyclist merely multiplies revolutions by their wheel’s diameter to derive an accurate measure of distance traveled, the pedal desk does not contain a traditional bicycle wheel and therefore any algorithm is the product of fantasy. That being said, there may be room for fantastical pedal desk journeys as part of a menu of motivational challenges designed to optimize adherence; however, it cannot be considered an accurate measure of behavior.

Power is a function of work and time. Although time is easy to track, work is a more difficult parameter to measure directly with a pedal desk. Since the pedal desk is intended to be used at a fixed and minimal level of resistance (thereby avoiding sweating during work and/or overuse injuries with extended use), the major factor contributing to work will be the user’s self-selected pace, measured in RPM. Currently power is not estimated with the pedal desk system; however, future improvements will incorporate user viewable estimates of power output in watts.

Likewise, energy expenditure is a parameter that cannot be easily measured only from duration and/or RPM tracked while using the pedal desk. Estimates can be informed by research of metabolic costs of pedal desk use while working and will likely require knowledge of the user’s sex, body mass, and age, for example. Again, we anticipate that any algorithm will be imperfect across individuals. That being said, day-to-day fluctuations in energy expended within an individual due to pedal desk use would be largely attributable to behavioral differences (i.e., duration used, revs/day) and presentations of calories burned may prove to be very motivational for individual users.

Limitations of the study presented herein included the relatively short duration over which pedal rate measurements were performed (i.e., approximately 20 min per person), and the device malfunctions leading to data loss associated with the Garmin Vector (n = 1) and pedal desk (n = 2). Moreover, it is possible that the presence of research staff in the testing area may have influenced participants’ self-selected pedaling rate. Strengths of this study include the use of automated data capture technologies which limit the potential for data recording and entry errors, and an assessment protocol which simulated conditions likely to be encountered in an actual working environment. Additionally, the described sample was drawn from a population of workers employed in a sedentary work environment, typical of many office settings for which the pedal desk may have its greatest potential impact.

This was a controlled study of pedal desk use parameters at a self-selected pedaling pace performed during a simulated working experience. Participants were not provided any digital or other type of feedback. Minute-by-minute and trial level (mean of approximately 20 min per trial) RPM measurements from the Pennington Pedal Desk™ demonstrated a high degree of accuracy in comparison to those concurrently obtained using the Garmin Vector power meter’s accelerometer-based cadence sensor. In sum, the Pennington Pedal Desk™ provided valid RPM measurements during a simulated working experience.
